# Design Principles and Applications of Fluorescent Kinase Inhibitors for Simultaneous Cancer Bioimaging and Therapy

**DOI:** 10.3390/cancers16213667

**Published:** 2024-10-30

**Authors:** Ab Majeed Ganai, Eirinaios I. Vrettos, Stavroula G. Kyrkou, Vasiliki Zoi, Tabasum Khan Pathan, Rajshekhar Karpoormath, Penelope Bouziotis, George A. Alexiou, George A. Kastis, Nicholas E. Protonotarios, Andreas G. Tzakos

**Affiliations:** 1 Department of Chemistry, Section of Organic Chemistry and Biochemistry, University of Ioannina, 45110 Ioannina, Greece; ganaimajid66@gmail.com (A.M.G.); eirinaios.vrettos@stjude.org (E.I.V.); stavroylakyrkoy@gmail.com (S.G.K.); tabasumkp@gmail.com (T.K.P.); 2 Neurosurgical Institute, University of Ioannina, 45110 Ioannina, Greece; vasozoi95@gmail.com (V.Z.); galexiou@uoi.gr (G.A.A.); 3 Department of Pharmaceutical Chemistry, Discipline of Pharmaceutical Sciences, College of Health Sciences, University of KwaZulu-Natal (Westville), Durban 4000, South Africa; karpoormath@ukzn.ac.za; 4 Institute of Nuclear and Radiological Science and Technology, Energy and Safety (INRASTES), National Center for Scientific Research “Demokritos”, 15310 Athens, Greece; bouzioti@rrp.demokritos.gr (P.B.); gkastis@academyofathens.gr (G.A.K.); nprotonotarios@academyofathens.gr (N.E.P.); 5 Mathematics Research Center, Academy of Athens, 11527 Athens, Greece; 6 Institute of Materials Science and Computing, University Research Center of Ioannina (URCI), Ioannina 45110, Greece

**Keywords:** bioimaging, conjugates, design principles, kinase inhibitors, theranostic agents

## Abstract

Kinase inhibitors are potent therapeutic agents in cancer treatment, but their effectiveness is frequently restricted by the inability to image the tumor microenvironment. To address this constraint, kinase inhibitor–fluorophore conjugates have emerged as promising theranostic agents, allowing for simultaneous cancer diagnosis and treatment. These conjugates are gaining attention for their ability to visualize malignant tissues and concurrently enhance therapeutic interventions. This review explores the design principles governing the development of multimodal inhibitors, highlighting their potential as platforms for kinase tracking and inhibition via bioimaging. The structural aspects of constructing such theranostic agents are critically analyzed. This work could shed light on this intriguing field and provide adequate impetus for developing novel theranostic compounds based on small molecule inhibitors and fluorophores.

## Simple Summary

This review highlights the recent advances in the development and application of dual function kinase inhibitors that also bear fluorescent properties (fluorescent kinase inhibitors), thus can be used as theranostics in the field of cancer. This is a rapidly growing field with significant potential for cancer therapy and diagnosis. This work mainly focuses on the key design principles that guide the development of these multifunctional compounds, emphasizing the integration of essential components such as the kinase cytotoxic warhead, the fluorophore, the linkers, and additional modular elements to enhance the efficacy of the final assembled compound. We anticipate this review to propel the advancement of this field by improving the understanding of the design principles and ultimately leading to the development of more effective tools for the concurrent diagnosis and treatment of cancer.

## 1. Introduction

Cancer is persistently one of the leading causes of death worldwide, responsible for nearly 20 million new cases in 2022 and almost 10 million deaths [1]. Unfortunately, these numbers are projected to rise to 30 million cases and more than 15 million deaths by 2040. For the past 10 decades, chemotherapy, surgery, radiation, or a combination of these have been the major contenders in cancer therapy [2,3,4,5]. Although these methodologies have aided in reducing the tumor burden, they generally do not provide a comprehensive therapeutic approach with high predictability for achieving long-term remission. Furthermore, the combinatorial utilization of anticancer agents is often invoked during chemotherapy, but it leads mostly to poor cancer suppression and severe side effects, mainly due to nonspecific drug targeting. The type of drugs used, along with the dosage and treatment frequency, can influence how radiation therapy and chemotherapy impact the innate and acquired immunity of cancer patients—both of which are crucial for defending against pathogenic threats [6]. The major disadvantage of the current cancer treatments is their tendency to affect normal cells, thereby crippling the host’s immune system. Thus, there is an unmet need for targeted therapeutic approaches, where the selective localization of the toxic warheads in malignant tumor sites can be boosted. Kinase inhibitors [7], small molecular weight compounds that target multiple receptors [8,9,10,11,12], are considered indispensable members of the targeted therapy approach [13].

Receptor tyrosine kinases (RTKs) are key targets for tyrosine kinase inhibitors due to their involvement in signal transduction and other cellular processes that drive cell proliferation and tumor growth. RTKs have been categorized into different families, including Type III RTKs (PDGFR, FLT3, and C-Kit), which are highly expressed in several cancer types, like breast or lung cancer [14,15]. All RTKs comprise three major parts: an intracellular tyrosine kinase domain, a transmembrane region, and an extracellular ligand binding domain [16]. RTKs’ abnormal activation in cancer has been closely correlated to the upregulation of the signaling of the phosphoinositide 3-kinase (PI3K) pathway [17]. When an appropriate stimulus, including vascular endothelial growth factor (VEGF) or epidermal growth factor (EGF), binds to the extracellular domain of RTKs, dimerization and phosphorylation of the intracellular tyrosine kinase domain occurs, leading to the engagement of PI3K to the plasma membrane [18]. Following this event, the PI3K/Akt cascade is activated, starting with the phosphorylation of PIP2 to PIP3, resulting in the recruitment of protein kinase B (also known as Akt) and phosphoinositide-dependent protein kinase 1 (PDK1) to the plasma membrane. Phosphorylation of the Akt by another protein kinase, known as a mammalian target of rapamycin complex 2 (mTORC2), leads to its activation and consequently to the activation of important target proteins that control cell proliferation, survival, and resistance to therapy [16,19]. Since the PI3K/Akt pathway is one of the most frequently dysregulated pathways in cancer, and because hyperactivation of its major components has been at least partly attributed to RTKs’ abnormal activation, research has nowadays shifted its focus to identifying novel tyrosine kinase inhibitors as potent medicines. 

Tyrosine kinase inhibitors can inhibit protein phosphorylation, which is responsible for transferring the signals intracellularly to regulate cell proliferation, survival, migration metabolism, and growth response to stimuli, and therefore, it plays a crucial role in anticancer activity [20]. Kinase inhibitor-based targeted therapy selectively identifies and damages specific types of cancer cells or tissues, sparing normal cells. This type of cancer treatment has greatly improved the quality of tumor manipulation, lessened the unwanted side effects, and is generally well tolerated in patients with advanced cancer progression and/or poor prognosis [21]. Notably, 62 kinase inhibitors had been approved by the U.S. Food and Drug Administration by February 2024 [5] including dasatinib [22,23], dabrafenib [24,25], sorafenib [26,27], sunitinib [28,29], and many more [5]. 

Besides the traditional targeted anticancer therapies, including chemotherapeutic drugs and kinase inhibitors, multiple innovative therapeutic approaches have emerged over the past decades. Along these lines, targeted therapy using theranostic agents that promote the selective and concurrent diagnosis and therapy of malignant tumors is of high relevance. Targeted theranostic agents usually consist of a fluorophore, an anticancer drug, and a tumor-homing element, tethered via various linkages [30]. The classic fluorophores that were used heavily in the past years included coumarin, anilinonaphthalene-sulfonic acid derivatives, dansyl amine, 4-*N*,*N*-dimethylamino-1,8-naphthalimide, prodan derivatives, Nile red, BODIPY, fluorescein, and rhodamine. The new era of theranostic agents consists of NIR (near-infrared) dyes that can be categorized based on their emission wavelength as NIR-I (700–1000 nm) and NIR-II (1000–1700 nm). These include polymethine cyanine dyes, dicyanomethylene-based dyes, squaraines, etc. NIR dyes offer several advantages over traditional fluorophores, including higher spatiotemporal resolution, improved signal-to-background ratio for imaging, and greater tissue penetration depth.

**Figure 1:**
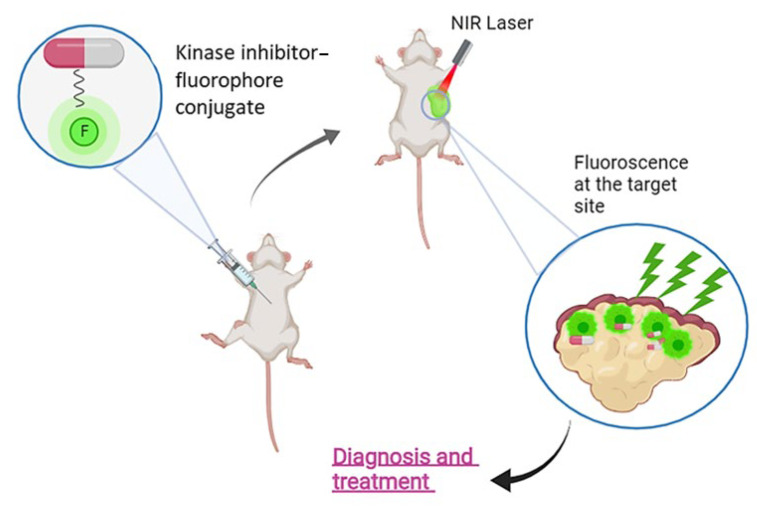
Example of fluorescence-guided diagnosis and therapy using fluorescent kinase inhibitors.

Combining kinase inhibitors with diagnostic modalities into a single entity could offer a dual-functional approach, enabling simultaneous cancer treatment and diagnosis. This strategy relies on the covalent conjugation of small molecular weight inhibitors to fluorophores via various linkers, to produce fluorescent kinase inhibitors, able to selectively visualize and eliminate cancer cells in a concurrent manner (Figure 1). This review focuses on the principles governing the design of fluorescent kinase inhibitors which can be utilized against the menace of cancer. An extensive analysis of their constituents (fluorophore, inhibitor, linker, and additional elements) is presented, followed by several breakthroughs in this field, according to the current literature. Special focus is placed on the design principles that guide the development of fluorescent kinase inhibitors. The basic structural elements required to formulate the architecture of fluorescent kinase inhibitors that include the kinase inhibitor, the fluorophore and the linkers that will connect these elements are also elaborated., A thorough analysis is provided on the selection criteria for the linkers that will tether the kinase inhibitor warhead to the fluorophore as well as additional components that could enhance the pharmacokinetics (e.g., solubility-enhancing moieties).

## 2. Design Principles and Analysis of Each Constituent

Fluorescent kinase inhibitors generally consist of a kinase inhibitor, a linker, and a fluorophore. However, some variants may also include additional components, such as moieties that enhance water solubility. The kinase inhibitor is used as the toxic warhead to eliminate the malignant tumor cells, the fluorophore (often a NIR dye) is used to enable the visualization of the tumor site, and the linker is used to tether the different elements and regulate the pharmacokinetic properties of the final conjugate. 

However, additional elements can be introduced to address specific weaknesses, such as off-target toxicity and low aqueous solubility. The following section will emphasize each constituent and rationalize the criteria for selecting them, with a particular focus on their design principles.

### 2.1. The Chemical Space of the Kinase Inhibitors

Protein kinases are defined by their ability to catalyze the transfer of the terminal phosphate group of ATP to certain substrates, which usually contain a serine, threonine, or tyrosine residue. Kinases have different structures, but they all consist of an activation loop, which is important in monitoring the kinase activity. The activation loop has different conformations with catalytically competent sites, usually phosphorylated, and an ‘inactive’ conformer site where the activation loop blocks the substrate binding site. Kinase inhibitors are mostly ATP competitive and bind to the activation site of ATP, inhibiting the protein phosphorylation and thus preventing cell proliferation. Certain representative kinase inhibitors are listed in Table 1, categorized according to their target protein. 

**Figure 2:**
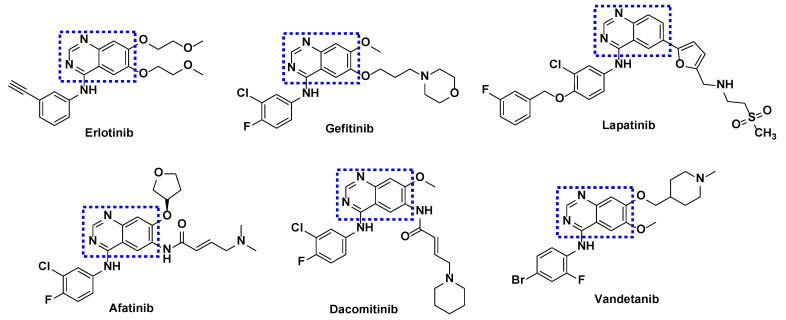
Quinazoline-based kinase inhibitors approved by the FDA for the treatment of different types of cancers. The quinazoline group is highlighted with a dotted blue line.

In addition to other core moieties found in kinase inhibitors, special attention should be given to the quinazoline group. This group has been extensively utilized in several FDA-approved EGFR kinase inhibitors (Figure 2) [31,32].

When designing a kinase inhibitor-based theranostic agent, the selection of the appropriate inhibitor should be guided by specific requirements. For instance, the inhibitor should bear the appropriate conjugation site (e.g., -COOH, -OH, -NH_2_) that could be utilized for its conjugation with a fluorescent dye or a linker. If the inhibitor does not possess the desired conjugatable site, a relevant analog can be sculpted in certain cases, but it must undergo full validation in both in vitro and in vivo settings to ensure its efficacy. Such an example was described by Mubarak et al. [33], where a conjugatable site (-COOH) was incorporated within the structure of sunitinib after the replacement of the *N*,*N*-diethyl- moiety. The free -COOH could be potentially utilized to conjugate a hydroxyl- or amine- containing fluorescent dye. Along these lines, similar procedures could be employed to produce various kinase inhibitors bearing the preferred conjugatable groups, provided that they do not disturb the interactions of the kinase inhibitor with its target protein. Usually, scientists prefer to exploit an already known kinase inhibitor in order to avoid the laborious synthesis of new analogs and the consequent in vitro and in vivo validation experiments. For this purpose, the crystal structures of numerous kinase inhibitors and their related target domain exist in data banks, and they should be employed to predict the appropriate conjugation site prior to the syntheses. For example, the crystal structure of dasatinib (kinase inhibitor) bound to its target (ABL kinase domain) suggests that the hydroxyl group of dasatinib points out of the binding site of the target and, hence, it could be modified without mitigating its inhibition potency [34].

In addition, another aspect that should be taken into consideration when selecting the appropriate kinase inhibitor is the type of cancer to be targeted. Each kinase inhibitor exhibits optimal efficacy against certain cancer types, and thus, these data should guide the kinase inhibitor selection. For instance, osimertinib has shown significant activity against mutated non-small cell lung carcinomas (NSCLCs) and could be one of the leading focuses for such types of cancers [35]. However, osimertinib should not be overlooked for other types of cancers, as it may have significant potency that may have not been unveiled yet.

### 2.2. Selecting the Fluorophore

**Figure 3:**
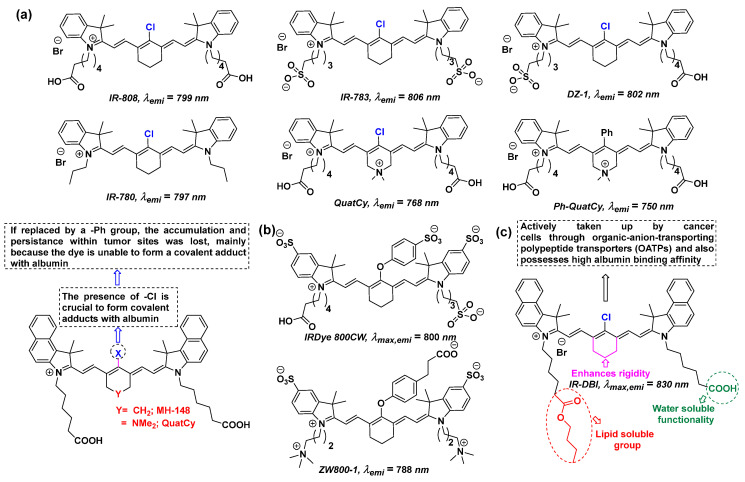
(**a**) Cyanine-based fluorescent dyes bearing either a meso-Cl (highlighted in blue) or a -Ph group. (**b**) Two cyanine derivatives used in clinical trials for fluorescence-guided surgery. (**c**) A multimodal therapeutic NIR dye containing a meso-Cl functionality [41].

Selecting a fluorophore with excellent photophysical properties is essential when designing a fluorescent kinase inhibitor, and cyanine dyes represent a promising option due to their favorable characteristics [36,37,38]. The following section is meant to describe some current breakthroughs in cyanine dyes [39], as the majority of previously reported kinase conjugates bear a cyanine dye core. The cyanine molecule features a conjugated π-electronic system and a push–pull structural element, which both contribute to its strong fluorescence and tunable photophysical properties. Heteroatoms, such as oxygen or nitrogen, act as electron donors (push component), while electron-withdrawing groups, such as -NO₂, function as electron acceptors (pull component). This push–pull configuration enhances the molecule’s electronic properties and fluorescence. It should be noted that nitrogen constitutes a better donor than oxygen because of its smaller electronegativity. If nitrogen has used its lone pair, it then behaves like an electron-withdrawing group [40]. In 2021, Syed Muhammad Usama and co-workers [41,42] summarized fluorescent heptamethine cyanine-7 (Cy-7) dyes, which have exceptional accumulation and persistence properties because of their in vivo covalent binding to albumin. The structures of such fluorescent cyanine dyes with their maximum emitted wavelengths are presented in Figure 3a. It was found that the meso-Cl group [41,42,43] is crucial for the dye to accumulate and reside longer in tumors, which is not the case with indocyanine green (ICG) because of the unavailability of the -Cl group. To confirm the importance of meso-Cl, dyes with different functionalities to a Cl group were tested, and it was evident that they did not produce a covalent adduct with albumin. The QuatCy dye derivative (Figure 3a), bearing a meso-Cl, formed covalent adducts with thiol-containing proteins other than albumin much faster than IR-808, because of the presence of a more electrophilic meso-carbon. However, dyes like IRDye 800CW and ZW800-1 (Figure 3b) are used in clinical trials as NIR contrast agents, although they do not contain a meso-Cl. Certain cyanine dyes can be used as treatment options in photothermal therapy (PTT) and photodynamic therapy (PDT) [44]. Along these lines, the Chunmeng Shi group [45] found a derivative of ICG (IR-DBI) with multimodal therapeutic activities including PDT and PTT (Figure 3c). The structural modification in IR-DBI seems to facilitate the binding to albumin, to form a dye–albumin complex that exerts a preferential accumulation and persistence at tumor sites via the enhanced permeability and retention effect. The released IR-DBI was taken up by the cancer cells via organic-anion-transporting polypeptide transporters and was selectively accumulated in the mitochondria, due to its lipophilic cationic nature. Apart from cyanines, other fluorescent chemotypes can also be used for conjugation with kinase inhibitors, including BODIPY, phthalocyanines, Alexa-532, and many more, depending on the specific requirements.

In addition to the photophysical properties, the choice of a fluorophore can also depend on its available conjugation sites, as is similarly considered for kinase inhibitors. The literature provides a pool of fluorophores with a wide variety of conjugation sites that can be selected based on specific requirements. Furthermore, existing fluorophores can be modified to incorporate the desired functional groups, a process that is generally simpler than the modifications required for kinase inhibitors.

Additionally, the selection of a fluorophore can be guided by the intended application of the final kinase inhibitor–fluorophore conjugate [43]. For instance, if the final conjugate is expected to be used solely for in vitro assays, the selected fluorophore is usually relatively small in size, so as not to perturb the binding affinity/selectivity of the inhibitor. If the final conjugate is expected to be utilized within in vivo applications, specific aspects must be considered: A higher molecular weight dye can be utilized (e.g., a NIR-II emitting dye), which is usually associated with deeper tissue penetration, lower signal-to-noise ratio and lower toxicity [46].

### 2.3. Types and Selection Criteria for Linkers in Kinase Inhibitor–Fluorophores

Despite its relatively small size compared to the rest of the theranostic agent, the linker is crucial in determining the bioactivity profile of the final kinase inhibitor–fluorophore conjugate. The linker must be carefully selected to optimize the pharmacokinetics and augment the delivery capacity of the kinase inhibitor to the target site while preventing a premature release. Additionally, the linker should not interfere with the binding affinity and selectivity of the kinase inhibitor towards its protein target. An inappropriate linker choice can lead to reduced efficacy or complete abolishment of the binding.

Additionally, the linker should be chemically/enzymatically resilient within the blood circulation to allow the conjugate to reach its kinase target intact and afford a spatiotemporal drug release within the tumor site. An example is discussed in the succeeding section whereby the conjugate **C8a** can selectively release the drug in the tumor site via a glutathione-mediated disulfide bond cleavage, increasing the efficacy of the utilized parent anticancer drug. The linker also plays a key role in keeping the dye out of the primary kinase domain site, which otherwise may interfere and result in diminished potency of the drug [47]. 

The most common linkers used in kinase inhibitor–fluorophore conjugates include amides and esters. These linkers are specifically designed to be cleaved in the cancer microenvironment, where high levels of amidases and esterases are present [48]. Additionally, ethylene glycol linkers are frequently employed among classic linkers to enhance the pharmacokinetic properties of the inhibitor. Another class of linkers that is continuously gaining attention involves linkers that are rationally designed to become cleaved under specific stimuli overexpressed in the tumor environment. For instance, certain bonds can be hydrolyzed selectively in the presence of the slightly acidic pH of the tumor microenvironment to release the active drug, while they are stable in the blood circulation [49]. These include imine, oxime, hydrazone, orthoester, acetal, and vinyl ether. A prior rational design could result in a theranostic agent that shows enhanced fluorescence intensity (derived from the utilized dye) when the kinase inhibitor is released into the tumor microenvironment. Along these lines, self-immolative linkers are occasionally utilized, as they offer a controlled drug release, triggered by various tumor microenvironment stimuli [50]. The trigger group that is used to detonate the on-demand drug release can be attached to the donor moiety of the dye, resulting in the quenching of its fluorescence until the conjugate reaches the tumor site.

### 2.4. Additional Structural Elements: Refining the Architecture of Kinase Inhibitor–Fluorophores

**Figure 4:**
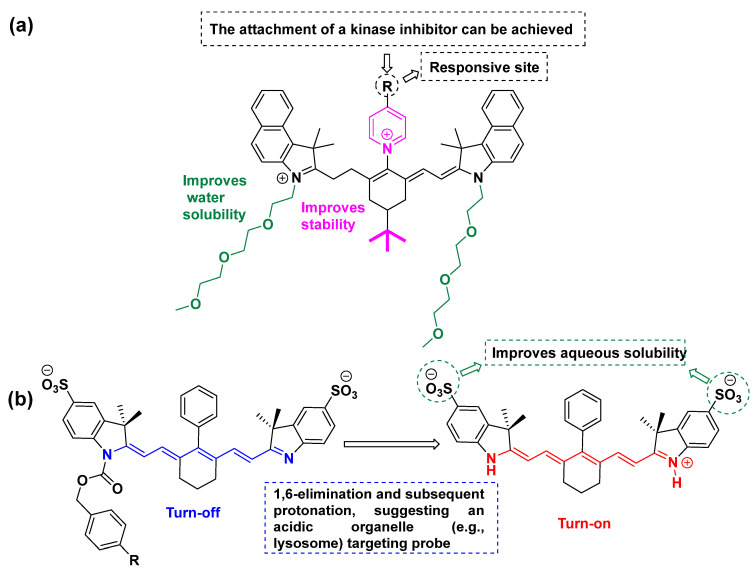
Water solubility enhancement by inserting different functional groups: (**a**) PEG and (**b**) SO_3_^−^.

Additional elements can be incorporated into the final conjugate to address various limitations, such as low water solubility, off-target toxicity, and insufficient chemical or enzymatic stability. Water solubility is a crucial parameter required to achieve the effective concentration of the drug in the target tumor area. The main issue of the majority of the drugs is their low aqueous solubility, which is closely associated with a low bioavailability. Various techniques are exploited to improve the water solubility of poorly soluble drugs, including physical and chemical modifications of the drugs. Specifically, this can be achieved through modification of their chemical structures with certain functionalities like -SO_3_^−^, -COOH, glycols, and morpholines. In addition, other techniques like particle size reduction, salt formation, solid dispersion, use of surfactant, encapsulation, nanoformulation, and complexation might also be employed. Along these lines, Juan Ouyang and co-workers developed an approach for the synthesis of various heptamethine cyanine-based NIR-II fluorophores with enhanced aqueous solubility and stability (Figure 4a) [51]. These were developed by introducing a pyridinium ring (on the top of the central cyclohexenyl group) and two PEG chains for solubility enhancement, and a tert-butyl group (on the central cyclohexenyl group) for stabilization. Recently, Usama and co-workers [52] reported a fluorogenic probe based on the heptamethine cyanine scaffold (Figure 4b). This probe bears two SO_3_^−^ moieties aiming to enhance its water solubility. The protonation of the nitrogen in an acidic medium triggers the enhancement of the fluorescence intensity and, hence, this agent represents a turn-on probe for acidic organelles like lysosomes. This compound could be utilized via its conjugation with a kinase inhibitor to develop a kinase inhibitor–fluorophore. 

**Figure 5:**
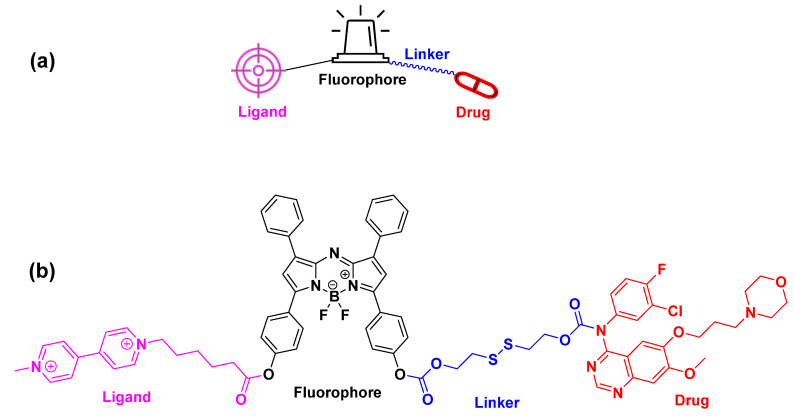
Incorporation of the tumor-homing polyamine to offer a selective accumulation of a theranostic BODIPY-gefitinib agent to the tumor site. (**a**) The general architecture of the fluorescent–drug conjugates with tumor-homing elements; (**b**) chemical structure of the BODIPY-gefitinib agent. The targeting element (polyamine) is colored purple, the fluorophore (BODIPY) black, the linker (disulfide bond) blue, and the cytotoxic and kinase targeting drug (gefitinib) red [56].

In addition to water-soluble moieties, tumor-homing elements can also be incorporated to improve the selectivity of the final kinase inhibitor–fluorophore conjugate. These elements enhance targeted delivery while reducing off-target effects and increasing therapeutic efficacy and can also function as stability/solubility enhancers (Figure 5a). Certain receptors are overexpressed or uniquely expressed on the surface of malignant tumor cells, representing appealing candidates for tumor targeting [49,53,54,55]. Targeting these receptors with specific biomolecules could shape a methodology to target the cancer microenvironment and has been extensively exploited for several years. Recently, Song et al. took advantage of the polyamine uptake system (PUS) by developing a polyamine-targeting agent of gefitinib [56]. This agent consists of gefitinib (drug), disulfide bond (linker), BODIPY (fluorophore), and polyamine (tumor-targeting element) and is used to treat non-small cell lung carcinoma. The conjugate binds to the PUS by the polyamine ligand, thus leading to its accumulation within the solid tumor (Figure 5b).

## 3. Representative Examples of the Architecture of Kinase Inhibitor–Fluorophores

The aforementioned structural components can be freely combined to create a single chemical entity consisting of a kinase inhibitor (toxic warhead), a fluorophore (visualization modality), necessary linkers (e.g., self-immolative linkers), and additional elements such as triggers or moieties that enhance solubility and stability. Representative examples of the current literature are presented in the following section and the design principles governing the selection of each constituent are extensively analyzed for each case.

### 3.1. Dasatinib-Based Conjugates

**Figure 6:**
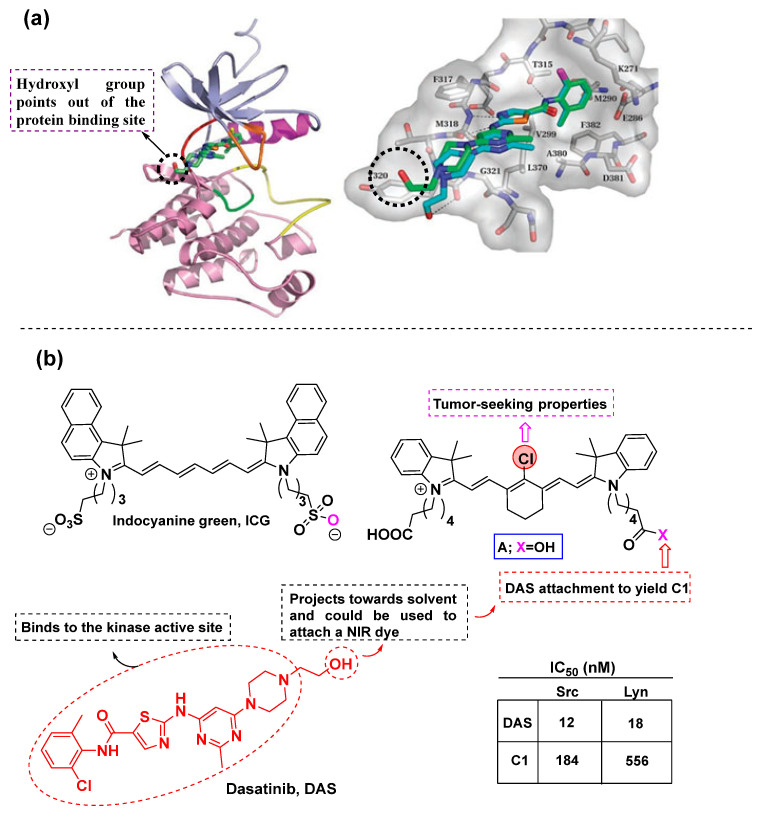
Dasatinib-based conjugates after rational design. (**a**) Crystal structure of dasatinib complex with ABL kinase, with the hydroxyl group pointing out of the kinase cavity [34]. (**b**) The structure of the fluorescent dasatinib-based analog **C1** utilized against glioblastoma and liver cancer [57,58].

Dasatinib is a kinase inhibitor approved for the treatment of certain types of leukemia. In addition to its efficacy in leukemia, it has shown potent activity against other cancers, including glioblastoma, the most aggressive form of brain tumor. Based on computational studies, dasatinib is known to bind to multiple conformations of the ABL kinase [34], where the hydroxyl group projects towards solvent and could probably represent a site to tether an NIR dye (Figure 6a,b). Based on its binding mode and ongoing clinical trials against glioblastoma, Kevin Burgess’s group [57] designed a cyanine-based dasatinib conjugate against glioblastoma and evaluated it in in vitro and in vivo settings. Based on the computational studies, the authors decided to conduct a direct conjugation with the cyanine dye **A** on the hydroxyl group of dasatinib, to develop the final conjugate named **C1**. The meso-Cl was not used due to its tumor-homing properties, as its binding to albumin could enhance its population within the tumor site. This was confirmed after comparing **C1** with ICG (Figure 6b) which does not bear the meso-Cl. Notably, the conjugation mode did not perturb the absorption and emission properties of the parent dye, as the conjugate **C1** absorbed (796 nm) and emitted (815 nm) within the same spectral region. The IC_50_ values of **C1** against Src and Lyn kinases were determined, and it was recorded that **C1** displayed higher IC_50_ values relative to the parent drug dasatinib (Figure 6b). Although the conjugate **C1** perturbed the affinity of the kinase inhibitor towards the studied kinases, significant binding was retained. After evaluating the in vitro cell viability of the synthesized conjugate, the authors aimed to determine its cellular uptake mechanism. Glioblastoma cells (U87) were treated with **C1**, and the results indicated that **C1** localized predominately in mitochondria due to its lipophilic and positively charged moieties. Finally, in vivo imaging in nude mice revealed the localization of **C1** in tumor sites for extended periods (~72 h). In a similar work, Kevin Burgess’s group [58], used the same conjugate **C1** against liver cancer cells (HepG2). Similarly, it was observed that **C1** displayed higher IC_50_ values in comparison with the parent drug. **C1** efficiently suppressed the viability of the HepG2 cells in a more efficient manner than plain dasatinib at the same concentration and also prevented their regrowth. **C1** further proved to be cell permeable and also to localize in mitochondria. 

**Figure 7:**
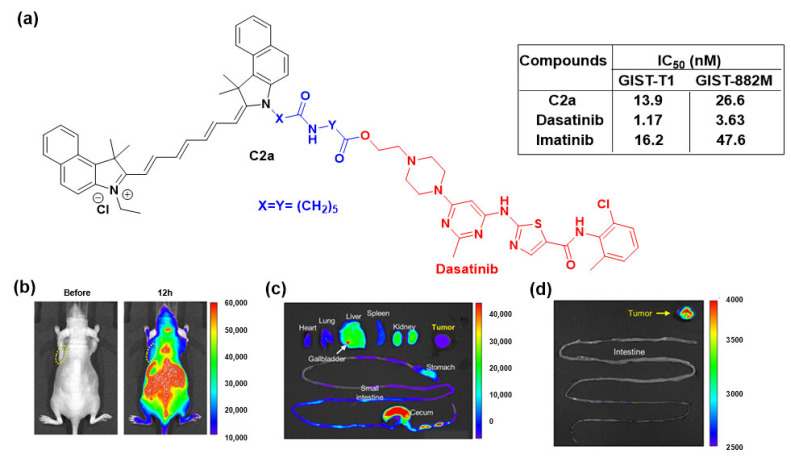
Dasatinib-based conjugates. (**a**) Fluorescent dasatinib-based conjugate **C2a** utilized against GIST cancer; (**b)** In vivo fluorescence imaging pattern of GIST-T1 xenografted mice treated with conjugate **C2a** (10 mg/kg injected intravenously) and fluorescence images acquired before and 12 h post-injection. The yellow dashed circles correspond to the tumors; (**c**) The ex vivo fluorescence images of the heart, lung, liver, gallbladder, spleen, kidneys, stomach, small intestine, cecum, and tumor acquired 48 h after injection of **C2a** (10 mg/kg); (**d**) The ex vivo imaging pattern of the tumors and intestines after washing with saline. The tissues were collected from mice 48 h after injection with **C2a** (10 mg/kg) and images were acquired after washing [59]. The dye is colored black, the linker blue, and the inhibitor (dasatinib) red.

It is known that gastrointestinal endoscopy is not able to effectively differentiate gastrointestinal stromal tumors (GISTs) from other subepithelial lesions. Therefore, the need for the development of pronounced treatments that could target a specific tumor tissue is critical. In the late 2020s, Fujimoto et al. [59] described a NIR-based conjugate of dasatinib, designated **C2a**, targeting GISTs. It was found that **C2a** visualizes both GIST-T1 and GIST-882M cells with moderate to good antitumor activity (Figure 7). Notably, the conjugate **C2a** displayed in vivo fluorescence signals in tumors with a high signal-to-noise ratio (SNR) ratio. Such a pattern of in vivo fluorescence imaging of GIST-T1 xenografted mice treated with **C2a** is shown in Figure 7b, in which **C2a** (10 mg/kg) was given intravenously and fluorescence images were acquired before and 12 h post-injection. The fluorescence images of various organs were acquired, as indicated in Figure 7c,d. In this study, fluorescence imaging was conducted in both subcutaneously xenografted mice and orthotopically xenografted rats to detect the accumulation of the conjugate in tumors. Therefore, this conjugate could operate as a useful architectural template when designing theranostic probes for GISTs. 

**Figure 8:**
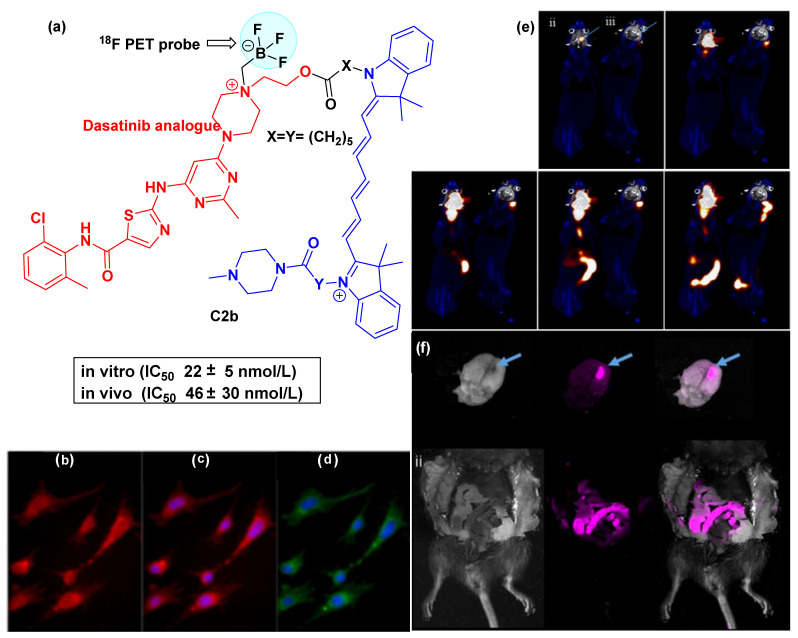
(**a**) The structure of the fluorescent dasatinib-based conjugate **C2b** utilized for PET imaging; (**b**) Fluorescence imaging of mBSG co-incubated with **C2b** shows fluorescence localization to glioma cells (15 min incubation); (**c**) similar distribution of staining in **C2b** fluorescence (red) and DAPI (blue) nuclei; (**d**) Cell mask plasma membrane stain (green)/DAPI(blue); (**e**) [^18^F]-1 delivery by CED to glioma at 15, 25, 40, 70, and 160 min. Blue arrows indicate glioma location. Mouse (ii) indicates successful CED delivery. Mouse (iii) indicates unsuccessful CED delivery. The correspondence of the imaging technique with the colors is as follows: PET(red)/CT(blue)/MR(grey); (**f**) ex vivo fluorescence analysis of [^18^F]-1 delivered by CED to the same mouse (ii). Fluorescence is represented in pink [60]. The dye is colored blue, the linker black, and the inhibitor (dasatinib) red.

In another study, the radioactive conjugate of dasatinib **C2b** (Figure 8a) was synthesized and delivered to murine orthotopic glioma by convection-enhanced delivery (CED) [60]. The ^18^F positron emission tomography (PET) and fluorescence imaging were used to track the entire drug delivery process. The localization of the fluorescence in the glioma cells was observed from the fluorescence imaging of mBSG co-incubated with **C2b** (15 min incubation) (Figure 8b)**.** Apart from this, a similar distribution of **C2b** fluorescence (red) and DAPI (blue) nuclei was observed (Figure 8c). It was observed that the conjugate exhibited in vivo nanomolar potency in cell viability assays, albeit slightly less effectively than the parent drug dasatinib (Figure 8) [59]. Figure 8e presents two mice that were infused with [^18^F]-1 by CED at time intervals of 15, 25, 40, 70, and 160 min. Glioma is indicated with blue arrows. Regarding the first mouse (symbolized by ‘ii’), CED delivery to glioma was successful, unlike the second mouse (symbolized by ‘iii’), where the delivery was unsuccessful. Importantly, PET imaging of [^18^F]-1 allows for real-time monitoring of the drug delivery to the tumor area. Figure 8f visualizes an ex vivo fluorescence analysis of [^18^F]-1 delivered by CED to the mouse (ii), followed by a PET scan.

### 3.2. Erlotinib-Based Conjugates

**Figure 9:**
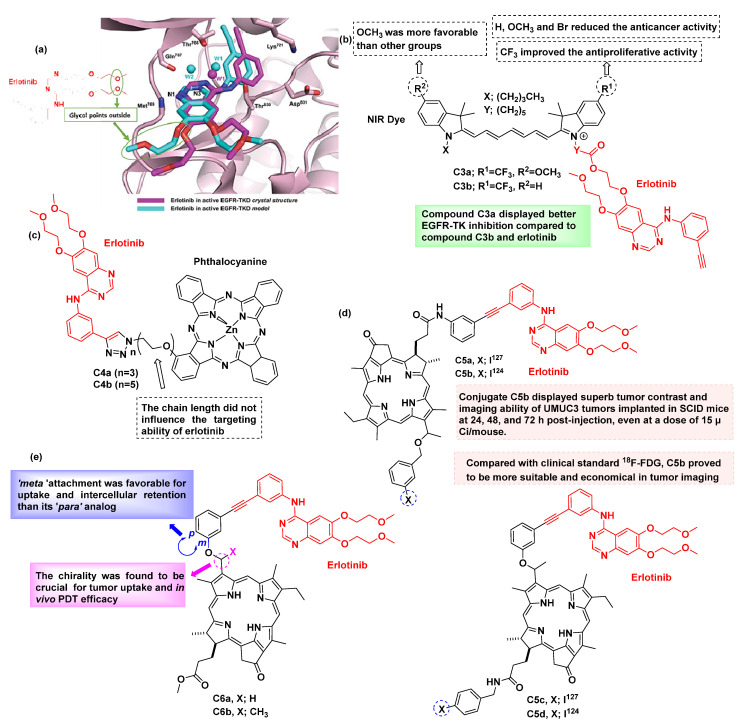
Erlotinib-based fluorescent inhibitors. (**a**) Erlotinib’s binding to active EGFR-TKD in the crystal structure and model [64]; (**b**) SAR study of NIR-based erlotinib conjugates **C3a** and **C3b** [61]; (**c**) Phthalocyanine–erlotinib conjugates **C4a** and **C4b** [62]; (**d**) Erlotinib conjugate **C5a** to **C5d** [63]; (**e**) Erlotinib conjugates **C6a** and **C6b** [47]. Erlotinib is colored red in all cases.

Erlotinib is an FDA-approved kinase inhibitor sold under the brand name Tarceva and is mainly used against NSCLC with mutations. It was observed that although the glycol part of erlotinib points out of the binding pocket, the modifications to the alkyne also retained the EGFR activity (Figure 9a). So, it is suggested that both parts of the erlotinib (glycol and alkyne) can be modified to achieve the desired results. Along these lines, in the year 2020, Xiaoguang Yang et al. [61] designed and synthesized several erlotinib derivatives conjugated with cyanine dyes (Figure 9b). The conjugation was achieved via the glycol moiety of erlotinib on the basis of computational studies. Molecular docking studies were also performed to confirm this hypothesis and revealed that the drug in the conjugate occupied the active site of the enzyme EGFR-TK (PDB: 1M17), whereas the NIR dye was found outside the protein cavity. The authors also performed structure–activity relationship (SAR) studies, and the key findings are illustrated in Figure 9b. Moreover, the cytotoxicity results revealed that most of the synthesized conjugates displayed better inhibition against A549, H460, H1299, and MDA-MB-231 cell lines in comparison with the parent drug erlotinib. It was observed that the conjugate **C3a** displayed higher EGFR-TK inhibition than erlotinib in the A549 cell line (Table 2) and also weaker cytotoxicity (38.6 μM) on human normal mammary epithelial MCF-10A cells. Therefore, the incorporation of a heptamethine cyanine dye into the glycol moiety of erlotinib could result in novel theranostic agents against NSCLC.

The current literature features numerous erlotinib conjugates formed through its alkyne moiety, which can be readily exploited in azide–alkyne click chemistry reactions. Using this type of click chemistry, Feng-Ling Zhang and co-workers [62] reported the design and synthesis of two erlotinib-based fluorescent conjugates (**C4a** and **C4b**) for simultaneous diagnosis and treatment via PTT (Figure 9c). The authors validated the selectivity of the conjugate for cancer cells overexpressing EGFR using confocal fluorescence microscopy. Both conjugates were evaluated for their subcellular localization and tested in vitro against HepG2 cancer cells, with IC_50_ values determined (Table 3). In vivo fluorescence imaging was performed in A431-bearing nude mice and revealed that the conjugate **C4a** accumulated in tumor tissues within 2.5 h. 

In another study, Ravindra K. Pandey’s [63] group evaluated the iodinated erlotinib-dye conjugates as dual bioimaging and therapeutic agents (Figure 9d). The developed conjugates were utilized as multifunctional photosensitizers for bladder cancer imaging and photodynamic therapy (PDT). The conjugates that were synthesized were evaluated for their anticancer activity (in vitro and in vivo) and were compared with the relevant conjugates without erlotinib. The stable iodinated erlotinib conjugates **C5a** and **C5c** displayed high EGFR targeting specificity. The PDT efficacies of **C5a** and **C5c** were significantly influenced by the topology of the conjugation between erlotinib and the remaining part of the conjugate, as evidenced by the higher efficacy of **C5a** compared to **C5c**. It was also found that **C5c** produced significantly fewer singlet oxygen species as compared with **C5a** in a biological environment and could be a possible reason for the difference in efficacies. Interestingly, it was found that the radioactive isotope **C5b** of **C5a** demonstrated admirable PET imaging ability. Therefore, this can be used uniquely in combination with **C5a** for the potential treatment of bladder cancers.

Another set of similar molecules was reported by Ravindra K. Pandey’s group [47], consisting of erlotinib conjugated to tetrapyrroles. Various analogs of the conjugates were evaluated, including the effect of chirality, the length of the linker, and the point of tethering between erlotinib and the dye. They found that these alterations affected the in-tumor cell specificity and in vitro PDT efficacy (Figure 9e). Furthermore, it was observed that the uptake and accumulation were higher for the conjugate **C6b** than for **C6a**, suggesting an important role of the chiral center in the accumulation. In this research, **C6b** was found to be the most potent analog for accomplishing tumor cell-specific accumulation.

### 3.3. Gefitinib-Based Conjugates

**Figure 10:**
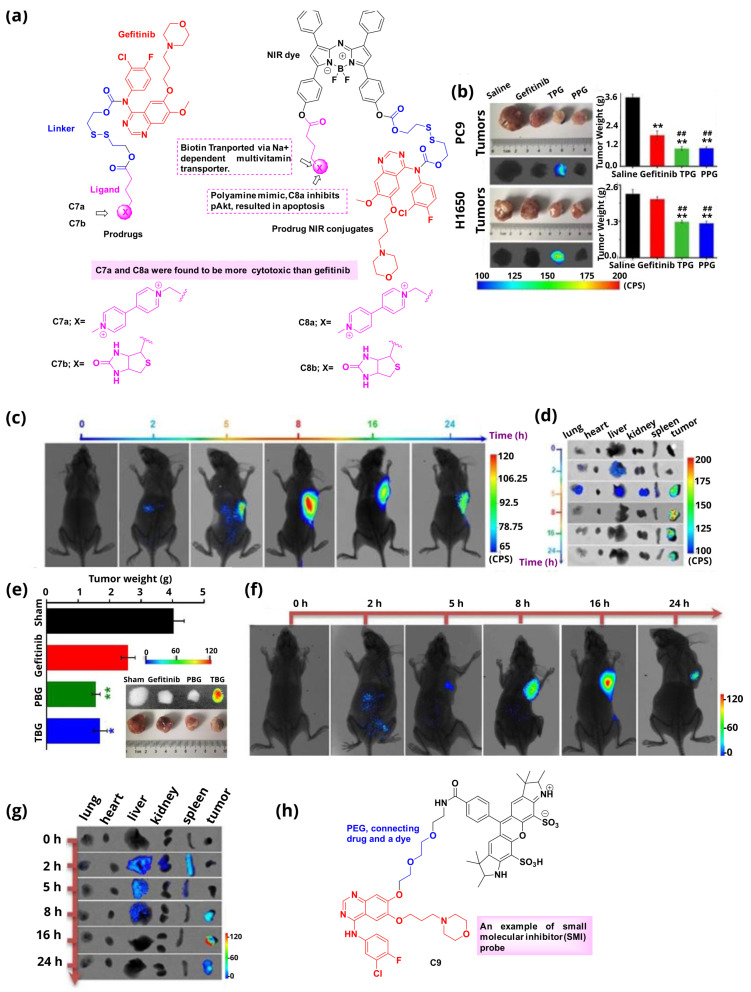
Gefitinib-based fluorescent inhibitors. (**a**) Structures of gefitinib-derived fluorescent conjugates **C8a** and **C8b**; (**b**) Tumor masses and fluorescence images of nude mice with PC9 cells and H1650 cells after treated with saline, Gefitinib, TPG-conjugate with the fluorophore, or PPG- conjugate without the fluorophore (0.5 mM in 0.2 mL, DMSO/saline, 1:1/*v*/*v*, qod. iv.) (n = 7 per group), ** *p* < 0.01 vs. control group. ## *p* < 0.01 vs. Gefitinib group; (**c**) Imaging of the subcutaneously implanted H1650 tumor xenografts of nude mice at 2, 5, 8, 16, and 24 h after tail vein injection of a single dose of 0.2 mL of TPG (DMSO/saline 1:1/*v*/*v*) (n = 3 independent experiments), (**d**) Images of the excised organs (lung, heart, liver, kidney, spleen) and tumors of the mice (n = 3 independent experiments) [56]; (**e**) Tumor masses and fluorescent images of nude mice bearing PC9 cells subcutaneous cancer xenografts were established and treated with saline, gefitinib, TBG, PBG (0.5 mmol/L in 0.2 mL saline, DMSO/saline (1/1, *v*/*v*), qod. i.v.) (n = 5 per group) * *p* < 0.05, ** *p* < 0.01; (**f**) and their xenografts for 2, 5, 8, 16, and 24 h after tail vein injection of a single dose of 0.2 mL of TBG (DMSO/saline (1/1, *v*/*v*)) (n = 5); (**g**) Images of the excised organs (lung, heart, liver, kidney, spleen) and tumors of the mice (n = 5 independent experiments); (**h**) Structure of gefitinib-derived fluorescent conjugates **C9** [65].

Gefitinib, sold under the brand name Iressa, is a medication used to treat various cancers, especially breast and lung cancers. It is an EGFR inhibitor that interrupts cellular signaling through the EGFR in target cells. The fluoro group of gefitinib is important for binding, so the reported molecules retain this group, and modifications are conducted in other parts of the core, like the -NH and -OCH_3_ functional groups of the drug. Along these lines, Song et al. [56,65] reported various fluorescent or non-fluorescent conjugates of gefitinib that consisted of the kinase inhibitor (gefitinib), a fluorescent dye (BODIPY) in the case of the fluorescent conjugates, a cleavable linker (disulfide), and different targeting ligands. The conjugates were evaluated against NSCLC (Figure 10a(i)). The conjugate **C8a** efficiently delivered the drug to cancer tissues after a glutathione-mediated disulfide bond cleavage without resulting in off-target toxicity, therefore increasing the efficacy of the anticancer drug [56]. Glutathione (GSH) is a thiol that plays an important role in cellular processes, and its expression levels in cancer cells are higher compared to normal ones [66,67,68,69,70]. The disulfide linker was chosen during the design so as to enable its selective cleavage in the tumor environment, with the consequent drug release, where the levels of GSH are enhanced. Furthermore, it was observed that the synergistic effect between the drug and ligand played a vital role in the observed enhanced efficacy of the conjugate, suggesting apoptosis via ligand-mediated Akt inhibition. The advantage of **C8a** over gefitinib is that it was selectively localized in the tumor cells (both sensitive and resistant to gefitinib) and the strong fluorescence derived from the dye lasted around 24 h post-injection. This suggests that the activity of the conjugate is based both on BODIPY and the tumor-targeting ligand. Specifically, experiments were performed on gefitinib-sensitive cells (PC9 cells) and gefitinib-resistant cells (H1650 cells) and the results indicated that this compound was able to inhibit H1650 cell growth. In vivo experiments (Figure 10b–d) pinpointed that the conjugate leads to the detection of lung cancer tumors within 4 h. Therefore, the authors describe a prodrug that significantly improves the pharmacokinetic properties of gefitinib, through the attachment of the polyamine-targeting agent, minimizing potential side effects. The resulting agent also absorbs radiation in the NIR region leading to the detection of tumors and on-demand drug release in real-time. The conjugate **C8b** consists of a biotin moiety, a disulfide linker, a NIR fluorophore, and gefitinib [65]. As in the previous example, the anticancer drug gefitinib is modified with a biotin-recognizable binder resulting in the prodrug termed PBG. PBG possesses improved pharmacokinetic properties, while it can also acquire imaging properties when combined with the near-infrared azo-BODIPY, leading to the fluorophore-TBG conjugate. The fluorescence of the conjugate was attained in the presence of high concentrations of GSH and could not be achieved in the presence of other stimuli including various amino acids, peptides, anions, metal ions, reactive oxygen species, and reactive nitrogen species, thus confirming the high specificity of the conjugate towards GSH. The experiments were performed on the human lung adenocarcinoma PC9 cell line and corresponding cancer-bearing nude mice. Saline, gefitinib, PBG, and TBG were injected via the tail vein every second day, and the measurements were obtained every two days for 28 days. Compared with saline, reduced cell proliferation was observed in both PBG and TBG, as shown in Figure 10e. The targeting ability of TBG was also examined in vivo, giving satisfactory results in accumulation in the tumor area after the first 8 h after injection (Figure 10f,g). It was also observed that the drug release depends on both GSH concentration and the Sodium-Dependent Multivitamin Transporter (SDMT) expression level. It can be concluded that the critical challenges faced with the present cancer therapies could be surpassed by the newly emerging theranostics, which can deliver diagnostics and therapeutics with high accuracy.

Hongda Wang’s group recently reported a small molecular inhibitor (SMI) probe (**C9**) for visualizing EGFR by utilizing gefitinib, a flexible linker, and a fluorescent dye (Figure 10h) [65]. The probe generated the highest labeling density and the smallest and most compact clusters, indicating its superiority toward accurate labeling of aggregated targets as compared to antibody and ligand probes. The conjugate demonstrated high specificity towards EGFR, suggesting that small molecule inhibitors (SMIs) can achieve significant target specificity. A key advantage of SMIs over antibodies is their ability to penetrate the cell membrane, allowing them to target intracellular compartments. This makes SMIs a straightforward method for fluorescence labeling of intracellular organelles without the need to disrupt the cell membrane. Additionally, dSTORM imaging revealed that SMIs provide a clearer spatial visualization of EGFR on the cell membrane compared to traditional total internal reflection fluorescence (TIRF) imaging. Additionally, multiple SMIs and especially kinase inhibitors possess high pharmacological activity [71]. The successful synthesis of such probes could be used to track the intracellular position of an SMI and unveil possible interactions between the SMI and related biomolecules. Thus, these findings might assist in revealing the exact mechanism of the interaction of drugs with their targets. With these advantages, SMI probes could serve as potential labeling agents in super-resolution fluorescence imaging.

### 3.4. Afatinib-Based Conjugates

**Figure 11:**
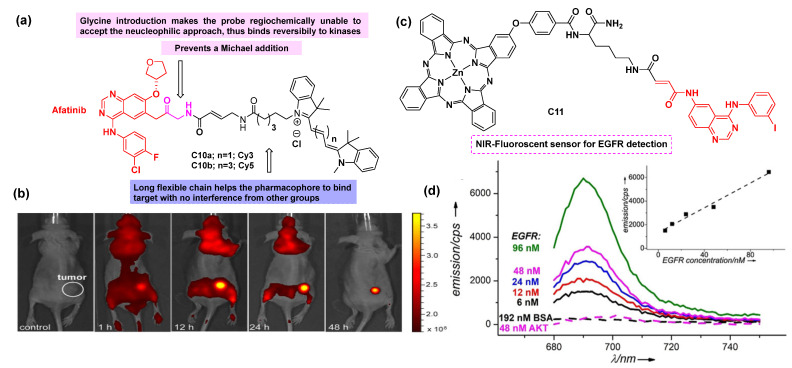
Afatinib-based fluorescent inhibitors. (**a**) Structures of afatinib-derived fluorescent conjugates **C10a** and **C10b**; (**b**) Fluorescence imaging obtained for **C10a**-treated xenografted mice for up to 48 h (λ_ex_ 540 ± 10 nm and λ_em_ 560 ± 20 nm) [72]; (**c**) Structure of the fluorescent conjugate **C11**; (**d**) Addition of increasing amounts of EGFR results in a turn-on fluorescent response of **C11** in aqueous conditions [73]. The inhibitor (afatinib) is colored red in both cases.

Qingzhi Gao’s group [72] reported small molecule fluorescent probes **C10a** and **C10b** consisting of a cyanine dye and a kinase inhibitor (afatinib) as efficient inhibitors for the detection of HER1/HER2 expression levels in cancer cells and in vivo tumor diagnostic imaging modality (Figure 11). Flow cytometry confirmed the reversible binding of the conjugate to kinases, as this was evident from the decreased signal intensity. The probes were unable to undergo receptor-mediated Michael additions, unlike the parent KI, because of the unavailability of the strategically positioned alkene. The synthesized conjugates **C10a** and **C10b** were evaluated through fluorescence imaging, flow cytometry, binding inhibitions, molecular docking, and in vivo tumor detection and demonstrated a high accumulation and cytotoxicity in xenografted tumors with a single dose. It was evident from ex vivo imaging that the fluorescence can be retained between 12-48 h post-injection in living mice, suggesting an efficient probe that could be further explored and tailored to achieve superior theranostic agents for HER1/HER2.

Evgueni Nesterov’s group reported the development of the conjugate **C11** for sensing EGFR tyrosine kinase, an essential target in cancer treatment (Figure 11c) [73]. The conjugate **C11** was used as an example of a small molecule anchor and was supposed to target EGFR, which was later justified. The probe’s turn-on mechanism was based on the aggregation/de-aggregation of phthalocyanine chromophores, which in turn depends on the selective binding of small molecules to their target biopolymer. Therefore, a turn-on fluorescence takes place with a high S/B ratio upon de-aggregation in a dark background of H-aggregated molecules without the need to remove unbound species. Thus, this approach makes it possible to design reliable turn-on NIR fluorescent sensors to detect specific protein targets present in the nanomolar concentration ranges (Figure 11d,e). 

### 3.5. Additional Examples of Fluorescent Drug Conjugates

**Figure 12:**
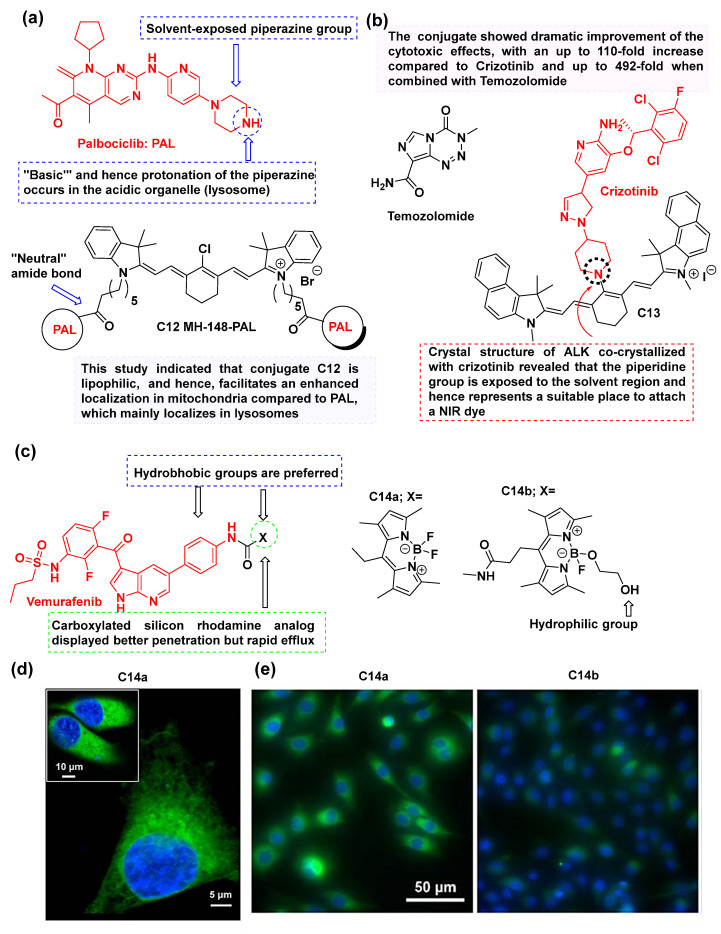
(**a**) The structure of MH-148-palbociclib conjugate **C12** [74]; (**b**) The structure of HMDA-based crizotinib conjugate **C13** [75]; (**c**) The structures of vemurafenib-based fluorescent conjugates **C14a** and **C14b** [76]; (**d**) High-resolution microscopy of **C14a** in A375 and SK-MEL-28 cells (inset); (**e**) In vitro imaging of **C14a** exhibiting prolonged cytoplasmic retention with minimal background fluorescence, in contrast to **C14b**, in SK-MEL-28 cells [blue: HOECHST 33342, green: BODIPY]. The fluorophores are colored black and the drugs in red all the examples [76].

There is an array of different fluorescent conjugates based on other KIs, including palbociclib [74], crizotinib [75], vemurafenib [76], ibrutinib [77], 5-bromobenzofuran-2-carboxylic acid [78], and nilotinib [79], some of which are described in the following section. In 2021, Euphemia Leung et al. [74] synthesized a conjugate, designated as **C12** (Figure 12a), after the conjugation of palbociclib with MHI-148 (NIR dye). The conjugation occurred in the piperazine group of palbociclib since it is solvent-exposed, pointing out of the kinase cavity. **C12** showed enhanced potency in inhibiting cell growth and viability as compared to plain palbociclib in breast cancer cell lines, and also in non-cancerous cells (Table 4). Palbociclib-treated cells illustrated a significant difference in G1 cell cycle arrest compared to treatments with the conjugate **C12**, confirming a different mode of action for the conjugate **C12**. **C12** also showed increased cytotoxic effects and strong inhibitory effects on proliferation, growth, and viability compared to MH-148, which did not show any inhibitory effects.

In 2019, Peter J. Choi and co-workers [75] described the synthesis and cytotoxic effects of a NIR-emitting crizotinib-based heptamethine cyanine dye conjugate **C13** (Figure 12b). The conjugate was evaluated in three different patient-derived glioblastoma cell lines and showed cytotoxicity in a nanomolar range of 50.9 nM (EC_50_) and antiproliferative activity of 4.7 nM (IC_50_) (Table 5). It was also found that the conjugate maintained the same mode of cellular uptake via organic-anion-transporting polypeptides (OATPs) as that of the parent heptamethine cyanine dye. The conjugate **C13** serves as an example of synthesizing a library of tyrosine kinase inhibitor-based NIR dye conjugates to afford potent fluorescent compounds to treat highly aggressive brain tumors. 

In 2023, Zhu and colleagues developed a glutathione (GSH)-activatable theranostic agent based on crizotinib, a cancer treatment drug, designed for dual imaging and therapeutic purposes in tumor cells. The conjugate demonstrated high specificity, selectively activating in environments with elevated GSH levels, a hallmark of tumor cells. The study confirmed its effectiveness in both cellular models and zebrafish, highlighting its capability for precise tumor cell imaging [80]. Again in 2023, Chen and his group developed albumin-decorated nanoparticles, containing a cyanine–crizotinib conjugate, which can concurrently visualize and treat c-Met-positive colorectal tumor cells [81]. The developed nanoparticles were able to selectively visualize tumor cells and upon laser irradiation, the conjugate exhibited phototherapeutic properties. 

The vemurafenib (BRAF^V600E^ inhibitor) conjugate **C14a**, consisting of a NIR dye, was found in the cytoplasm of A375 and A375R tumor cells, indicating a potent cytosol localization and retention of the conjugate [76] (Figure 12c). The BODIPY-derived conjugate (**C14a**) possessed the most intriguing properties as compared to the MayaFluor and carboxylated silicon rhodamine analogs. The conjugate **C14a** showed efficient penetration into the cytoplasm of melanoma cells with extended retention as compared to the conjugate **C14b**, which comparatively showed poor penetration and retention. This could be attributed to the more hydrophilic nature of the MayaFluor-derived conjugate as compared to the BODIPY-derived one, which has a hydrophobic cleft. The high-resolution microscopy of **C14a** in A375 and SK-MEL-28 cells is illustrated in Figure 12d along with its in vitro imaging which displayed prolonged cytoplasmic retention with minimal background fluorescence (Figure 12e). Properties such as subcellular localization, target specificity, and slow dissociation kinetics make it crucial for effectively visualizing the targets of vemurafenib. Furthermore, in vivo imaging confirmed that conjugate **C14a** accumulated with preferential localization in tumors that responded to vemurafenib, and its fluorescence was retained even after 24 h post-injection. In 2023, Sabrina Taliani and colleagues developed a cy5-based NIR fluorescent vemurafenib analog to study BRAF^V600E^ in cancer cells [82]. The scientists demonstrated that the specific conjugate could enter BRAF^V600E^ mutant cells, bind to its target with high affinity, and then inhibit MEK phosphorylation and cell proliferation.

The conjugate **C15** was obtained by combining BODIPY with the Bruton tyrosine kinase inhibitor (ibrutinib) to generate a single-cell diagnostic imaging agent while preserving its irreversible target binding [77]. The conjugate demonstrated significantly reduced inhibition (approximately 100-fold) against the purified BTK enzyme compared to the parent kinase inhibitor, ibrutinib. However, it showed excellent in vivo target localization, with the capability to measure drug distribution and target inhibition. In vivo tumor imaging of a representative mouse with a BTK-positive HT1080 tumor provided key imaging insights before, and at 2, 5, and 24 h post-intravenous administration of **C15**. Extensive drug accumulation was noted in all cells, persisting even at the 24 h time The conjugate was also detectable for a longer period (>24 h) inside cancer cells, indicating that an efficient irreversible binding of the drug persisted. These longer hours of retention and persistence will help in examining the BTK-related cell environment, thus opening a new window for BTK inhibitors with the fluorescent tag.

**Figure 13:**
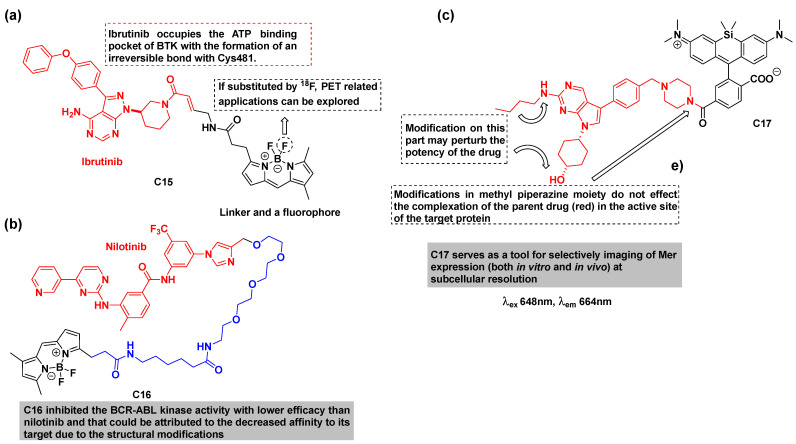
(**a**) The structure of ibrutinib-derived fluorescent conjugate **C15** [77]; (**b**) The structure of nilotinib-derived fluorescent conjugate **C16** [79]; (**c**) The structure of UNC2025-derived fluorescent conjugate **C17** [86]. The fluorophores are colored black and the drugs red in all the examples.

Nilotinib, sold under the brand name Tasigna, is used to treat chronic myelogenous leukemia (CML). This medication suffers from some resistance, which is the main disadvantage of some kinase therapies. Along these lines, Suresh V. Ambudkar’s group reported a nilotinib-based BODIPY conjugate as evidence for the transport of nilotinib and its fluorescent derivative **C16** by ATP-binding cassette (ABC) drug transporters (Figure 13b) [79]. ABC transporters are proteins that have been related to the detoxification of insecticides [83], multiple functions in reproductive tissues [84], transportation of photodynamic therapeutic agents by ABCG2 [85], and many more functions. 

The development of drug resistance in CML has been associated with the efflux of tyrosine kinase inhibitors by ABC drug transporters, which actively pump the drugs out of the cells using ATP as an energy source. This study aimed to unveil the TKI-ABC drug transporter interactions with Pgp and ABCG2 using the fluorescent conjugate **C16** to confirm the possible route of drug uptake and related drug resistance. It was observed that the total intracellular levels of **C16** in Pgp- and ABCG2-expressing cells were lower as compared to the cells that do not express such transporters, signifying that it is actively pumped out of these cells. This efflux of the conjugate was inhibited by specific inhibitors of Pgp and ABCG2 in both in vitro and ex vivo assays. These observations collectively suggest that the conjugate is pumped out of the cell via Pgp or ABCG2. Furthermore, both nilotinib and the conjugate **C16** inhibit Pgp and ABCG2 and also bind at the substrate binding site, but not at the ATP-binding site, of these transporter proteins.

UNC2025 is an ATP-competitive and highly orally active **Mer/Flt3** inhibitor with IC_50_ values of 0.74 nM and 0.8 nM, respectively. UNC2025 is >45-fold selective for MERTK relative to Axl (IC_50_ = 122 nM; K_i_ = 13.3 nM). UNC2025 exhibits an excellent PK property and can be used for the investigation of acute leukemia. With this knowledge, an imidazopyrimidine-based conjugate **C17** (MERi-SiR) was designed, synthesized, and studied for imaging of tyrosine kinase Mer (MERTK) [86]. The conjugate consists of UNC2025, a preclinical inhibitor of tyrosine kinase Mer, and silicon rhodamine carboxylate, an NIR-emitting fluorescent agent. Crystallographic studies suggest that methyl piperazine serves as an efficient site for attaching an NIR dye without significantly affecting the overall complexation (Figure 13c). It also suggests that the fluorochrome gets exposed to the solvent, thus preserving the compound’s interactions with the binding pocket of the Mer active site. Furthermore, the imaging results revealed that the conjugate **C17** accumulated in the cytoplasm on cells overexpressing Mer (SK-MEL-3 melanoma). The cytotoxicity results suggested that the conjugate showed reduced inhibition compared with the parent kinase inhibitor but co-localized with Mer in vivo. The conjugate demonstrated higher uptake and accumulation in Mer-expressing tumor-associated macrophages than in the tumor cells themselves, as revealed by the confocal microscopy of metastases in mice.

**Figure 14:**
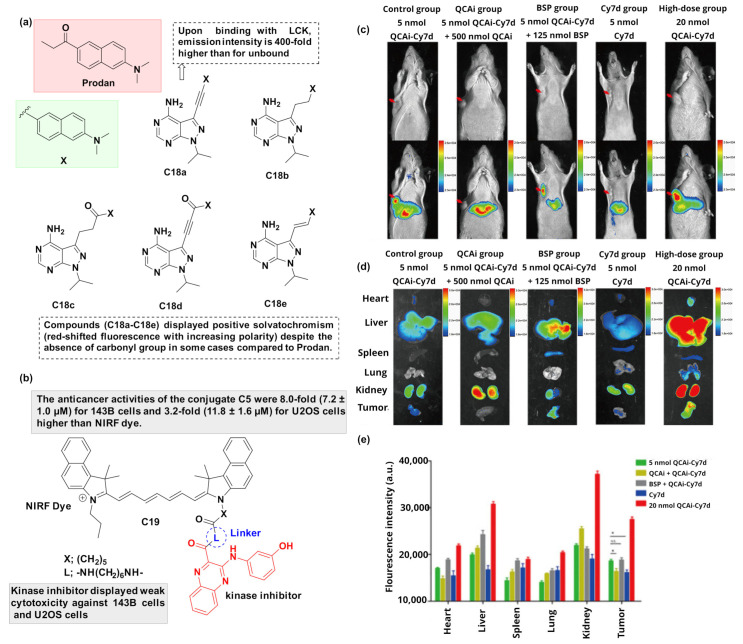
(**a**) The structures of prodan-derived fluorescent conjugates **C18a** and **C18e** [89]; (**b**) The structure of NIR dye-based PIM1 conjugate **C19** [91]. NIR fluorescence imaging and biodistribution of **C19** in vivo; (**c**) NIR imaging results of whole body at 48 h after the injection of the five different preparations (red arrow indicates tumors); (**d**) NIR fluorescence imaging results of organs and tumors at 48 h post-injection; (**e**) Graph presenting the fluorescence intensity of organs and tumors treated with different preparations (exposure time: 2 s). The drug is colored red, the linkers blue, and the fluorophores black.

Pyrazolopyrimidine and quinazoline scaffolds are important in drug discovery and their analogs have been explored as various kinase inhibitors [87,88]. Recently, Joakim Andréasson’s group reported pyrazolopyrimidine-derived prodan analogs (**C18a**–**C18e**) (Figure 14a) as fluorescent kinase inhibitors [89]. Polarity-based fluorescence probes such as prodan analogs have demonstrated excellent spectroscopic properties, including high fluorescence quantum yield and molar absorption coefficient, and excellent photostability [90]. It was observed that **C18a** and **C18c** displayed favorable fluorescent properties in aqueous solution and thus, they were evaluated for their ability to inhibit protein kinases. The two conjugates were initially tested against a panel of 65 kinases at 1 μΜ to screen their efficacy and selectivity. The conjugated analog **C18a** demonstrated strong inhibition against Aurora-A, Blk, and LCK, as compared to the non-conjugated **C18c**, where there was no apparent inhibition. Based on these encouraging results, **C18a** was further evaluated for cell-free IC_50_ assays against Aurora-A, Blk, and LCK and revealed moderate to good activity (IC_50_: Aurora-A: 222 nM, Blk: 554 nM and LCK: 124 nM). Finally, **C18a** was subjected to multiphoton imaging experiments in live cells and demonstrated a favorable cross-section for two-photon microscopy (TPM) experiments. Thus, it is believed that **C18a** could operate as an interesting molecular tool for real-time intracellular studies of LCK signaling.

In 2021, Xinzeyu Yi and co-authors [91] developed a NIR-based drug conjugate **C19** (Figure 14b) to target osteosarcoma, the most common malignancy of the skeletal system, associated with the overexpression of PIM1 kinases. It was observed that **C19** displayed targeted imaging and anticancer activities (greater than the parent inhibitor) without much toxicity. NIRF images of the entire body, major organs, and tumors were acquired (Figure 14c–e). The NIR fluorescence imaging results of the organs and tumors were acquired at 48 h post-injection. There was a reduction in the fluorescence intensity within a few hours after injection, potentially indicating that OATPs may contribute to early cell entry of the compound. In addition, cyanine dyes without a meso-Cl group, as used in **C19**, do not remain in tumor tissues for a long period because they are unable to form covalent adducts with free thiol-containing biomolecules, like albumin. Furthermore, the conjugate **C19** accumulated in mitochondria, and thus, this approach may suggest a way to design conjugates for simultaneous NIR-guided surgery and chemotherapy.

**Figure 15:**
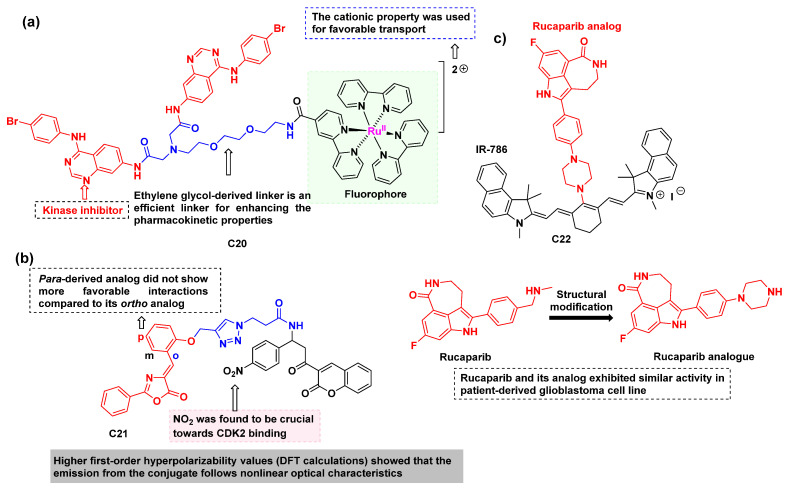
(**a**) The structure of the quinazoline-based Ru(II)-Bipyridine theranostic conjugate **C20** [92]; (**b**) The structure of the oxazolone–coumarin derived conjugate **C21** as a solid-state emitter [93]; (**c**) The structure of conjugate **C22** targeting three different patient-derived glioblastoma cell lines [95]. The drugs are colored red, the linkers blue, and the fluorophores black.

Rashid Ilmi and co-workers [92] reported a quinazoline-based fluorescent conjugate, designated **C20**, consisting of a Ru(II)-Bipyridine complex, an ethylene glycol linker, and a kinase inhibitor (Figure 15a). The theranostic agent **C20** combines EGFR inhibition with fluorescence imaging properties, and it was found to localize in mitochondria, suggesting that it acts as an EGFR optical probe. Therefore, these organometallic conjugates hold potential for further exploration to develop more potent theranostic agents for EGFR-overexpressing cancers.

Aranhikkal Shamsiya and Damodaran Bahulayan [93] reported several fluorescent derivatives of oxazolone–coumarin-based triazoles as anticancer agents (Figure 15b). Among the synthesized analogs, **C21** displayed the maximum calculated binding affinity with a binding score of 10.7 kcal/mol, suggesting that the nitro group enhances the binding affinity towards cyclin-dependent kinase-2 (CDK2). The experimental validation of the docking results was carried out using Western blot analysis using *b-actin* as an internal standard. The results indicated that the conjugate has a high potential to downregulate CDK2. This was supported by the fact that the active site of CDK2 comprises several amino acid residues that contain hydrophobic groups favorable to the formation of a tight ‘hydrophobic pocket’ in CDK2. The obtained binding energies suggest that the conjugate **C21** exhibits a strong hydrophobic interaction with the target CDK2. Consequently, downregulation of CDK2 was observed using Western blot analysis. Furthermore, the conjugate **C21** was subjected to anticancer evaluation against human cervical cancer cells (HeLa). The results indicate that the conjugate exhibits promising cytotoxicity against HeLa cells with an IC50 value of 25 mg/mL. This highlights the need for further studies to investigate its optical properties and efficacy against CDK2. In 2020, research exploring the kinase polypharmacology landscape of clinical PARP inhibitors revealed that niraparib and rucaparib inhibit DYRK1s, CDK16, and PIM3 kinases at clinically achievable, submicromolar concentrations [94]. Peter J. Choi and co-workers [95] designed, synthesized, and evaluated in vitro the activity of the rucaparib-based NIR-emitting conjugate **C22** (Figure 15c). It was found that the conjugate **C22** had a strong cytotoxic activity (EC_50_: 128 nM) against three different patient-derived glioblastoma cell lines. The synergistic effect of **C22** with the standard drug temozolomide (TMZ) for glioblastoma was observed, as evidenced by a two-fold reduction in the EC50 value, even in cell lines resistant to TMZ treatment (Table 6). Furthermore, the results suggest that **C22** has a high dependence on OATPs for their uptake into the tumor cells, similar to cyanine dye IR-786. The treatments based on cyanine conjugates of small molecules have a high effect on three different patient-derived glioblastoma cell lines and thus could be further explored to achieve the desired potent compound for various brain cancers. To further validate the conjugate against kinases, evaluations could be conducted with conjugates of rucaparib against various kinases such as DYRK1s, CDK16, and PIM3 kinases. 

**Figure 16:**
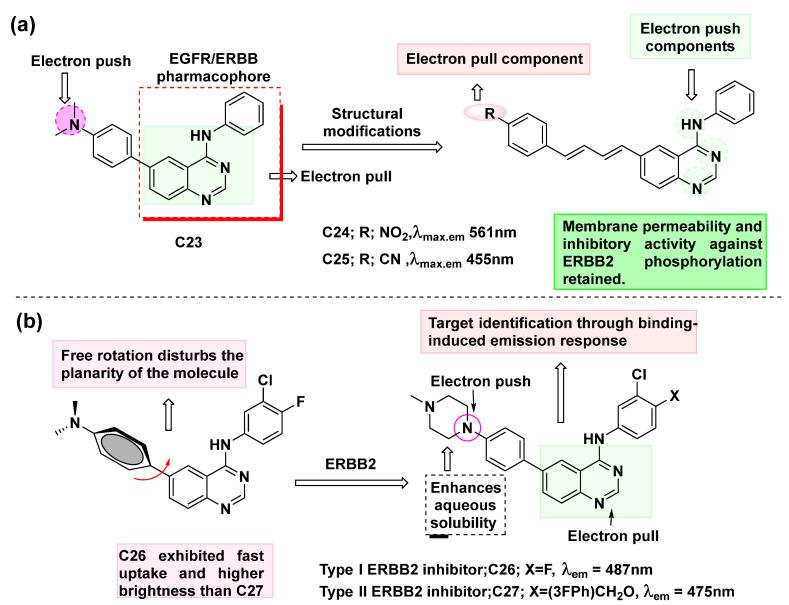
(**a**) The structure of fluorescent quinazoline-based analogs **C23–C25** [98]; (**b**) The structure of quinazoline-based fluorescent molecules **C26** and **C27** [97].

Besides the aforementioned examples, certain fluorescent inhibitors are not classified as conjugates because the fluorophore moiety is inherently part of their structure and not added post-conjugation. Notably, there are cases where the kinase inhibitor becomes fluorescent only upon binding to its respective target. James N. Wilson’s group [96,97,98] developed certain quinazoline-based fluorescent kinase probes as theranostic agents. Molecule fluorescence tuning [98] was performed by changing the extent of π-conjugation and by modifying auxochrome substitution (Figure 16a). It was found that the quinazoline moiety exhibited a dual character, acting as both an electron-donating and electron-withdrawing component, depending on the substitution pattern and the potential movement of the π-electron current (**C23** and **C24**). Moreover, the strongest electron-donating (push) and withdrawing groups (e.g., -dimethylamino, -cyano, and -nitro) produced high on/off ratios, indicating that they are desirable candidates for designing future fluorescent probes. The presence of a fluorophore arm at the 6-position of quinazoline hardly affects the ability of the fluorescent probes to operate as ERBB inhibitors. The resulting probes possessed low aqueous solubility, and this could be resolved by attaching hydrophilic groups to the fluorophore, based on the design principles described in the introduction part.

Similar types of molecules like **C26** were reported [97] and were found to ‘turn on’ when bound to kinases, suggesting a planner structure after binding the active site of the enzyme (Figure 16b). It was found that probe **C26** has a high affinity to identify ERBB2-overexpressing cells through a binding-induced emission response. The higher level of ERBB2 expression in BT474 cells permits improved binding of **C26**, which can be attributed to the higher emission intensity from BT474 cells compared to MCF7. These observations thus demonstrate that **C26** can stratify individual live cells by its dynamic response to activation. 

**Figure 17:**
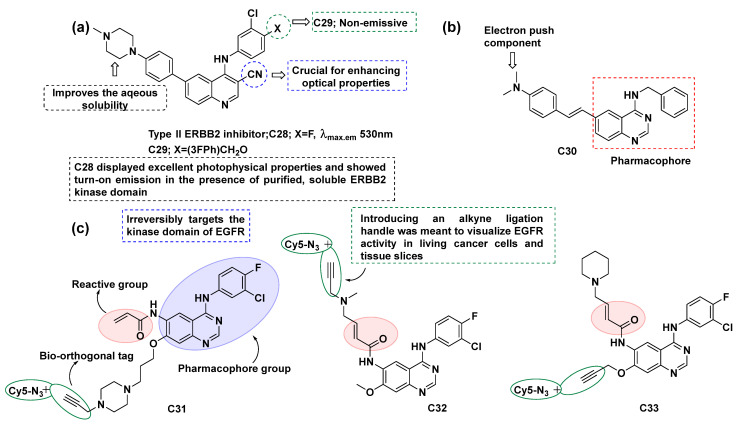
(**a**) The structure of cyanoquinazoline-derived fluorescent inhibitor **C28** [96]; (**b**) The structure of quinazoline-derived fluorescent inhibitor **C30** [99]; (**c**) The structure of quinazoline-derived fluorescent inhibitors **C31**, **C32** and **C33** [100].

In a continuation of earlier research, a 3-cyanoquinoline [96] core was investigated for its optical and biochemical properties (Figure 17a). It was found that the incorporation of this core improved the optical properties as compared to the previously reported molecules, suggesting the significance of the nitrile group in stabilizing the charge transfer excited state and red shift emission. Furthermore, **C28** demonstrated a moderate affinity for ERBB2 and was found to target the intracellular pool, permitting this fluorescent probe to operate as a reporter of the rapid dynamics of kinase internalization. 

**Figure 18:**
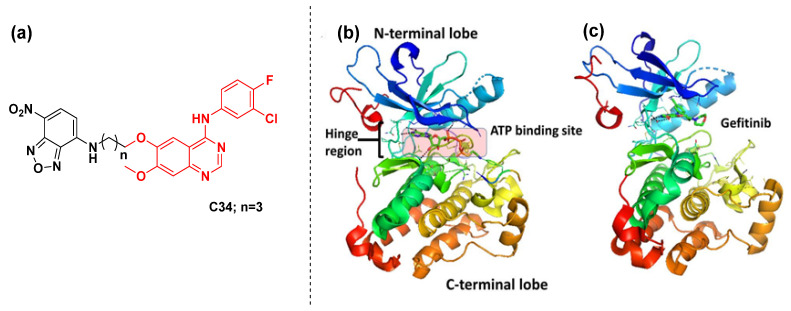
(**a**) The structure of quinazoline-derived fluorescent molecule; (**b**) Crystal structure of the kinase domain of EGFR with ATP binding site highlighted (PDB ID: 2GS6). TKIs of EGFR bind to EGFR in the ATP binding pocket, forming 1 to 3 hydrogen bonds to the hinge region; (**c**) EGFR kinase domain with gefitinib bound in the ATP binding pocket (PDB ID: 3UG2) [102]. The drug is colored red.

Renaud Sicard et al. [99] reported **C30** as a fluorescent reporter to investigate ERBB populations and their state of activation (Figure 17b). It was found that the synthesized fluorescent ‘turn-on’ probe targeted the ATP binding pocket of ERBB and enhanced emission was observed while bound, which could be due to the restricted geometry of the ERBB2 kinase domain. In 2022, Weimin Li’s group reported 4-anilinoquinazoline-derived molecules as clickable probes for the visualization of EGFR activity [100]. Three probes (**C31**, **C32**, and **C33**) were designed, synthesized, and evaluated against EGFR inhibition (Figure 17c). Probe **C31** was found to be the most potent analog, with the highest reactivity towards EGFR kinase with primary mutations: the IC_50_ values towards HCC827 and H1975 were 0.2 and 3.1 μM, respectively. Activity-based protein profiling (ABPP) was employed to visualize the protein activity and revealed that fluorescence labeling is specifically dependent on the clickable probes. Thus, probe **C31** may serve as a useful diagnostic tool and could improve the diagnosis of EGFR mutations and help in the EGFR-TKI therapeutic strategies. Jun Sheng’s group reported a few gefitinib-derived molecular probes as turn-on fluorescent ligands to make the visualization of EGFR protein possible (**C34**, Figure 18a) [101]. The crystal structure of the EGFR kinase domain complexed with tyrosine kinase inhibitors (gefitinib) is illustrated in Figure 18b,c. The fluorescence imaging and in vivo xenograft tumor imaging suggest that probe **C34** particularly responded to tumor cells overexpressing EGFR. The EGFR inhibition of the probe was evaluated in A431 cells, demonstrating that it retains its function as an EGFR inhibitor. These results suggest that probe **C34** could be used for fluorescence imaging of cells overexpressing EGFR and thus adds a fluorescent tag to the present therapy, which may help to understand the tumor and its environment accurately. 

#### Other Examples of Kinase-Based Theranostic Targeting Gliomas

**Figure 19:**
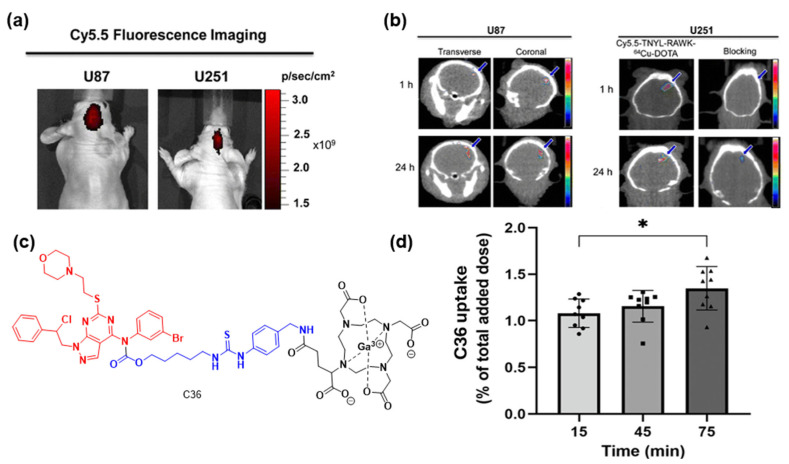
(**a**) Cy5.5 NIR optical imaging of U87-Luc and U251-Luc tumors 24 h after a tail vein injection of **C35**. (**b**) μPET/CT images of U87-Luc tumors (**left**) and U251-Luc tumors with **C35** and **C35** plus an excess of unlabeled **C35** (**right**) 1 h and 24 h post-injection [103]; (**c**) Structure of **C36**; (**d**) Cellular uptake of **C36** in U87MG glioblastoma cancer cells (experiments done in triplicate, * *p* < 0.05) [104].

Miao Huang et. al. developed **C35**, aiming to discover if it can be used as an imaging agent for μPET/CT and NIR imaging to treat orthotopic glioblastoma brain tumors [103]. **C35** consists of the EphB4-binding peptide TNYL-RAW, the radiometal chelator DOTA (1,4,7,10-tetraazadodecane-N,N′,N″,N‴-tetraacetic acid), which was utilized to chelate ^64^Cu, and the NIR dye Cy5.5. The authors initially utilized optical imaging (Figure 19a) and then μPET/CT (Figure 19b) to identify that U87-Luc and U251-Luc tumors can be efficiently visualized in mice. Therefore, it was supported that **C35** is capable of selectively binding to EphB4-expressing angiogenic blood vessels and EphB4-expressing tumor cells, rendering it an appealing bioimaging agent for both PET/CT and optical imaging of glioblastoma. 

Chiara Vagaggini et. al. developed the theranostic prodrug **C36** (Figure 19c) to selectively target and eliminate glioblastoma tumors [104]. **C36** consists of SI306, a potent inhibitor of Src (non-receptor tyrosine kinase), a linker, and DOTA chelated with radioactive ^68^Ga. First, the authors evaluated the ADME properties and the biological profile of the theranostic prodrug before the chelation of ^68^Ga, showing appealing properties and effective reduction in the cell viability of GL261 and U87MG glioblastoma cell lines. Then, the authors validated the effective time-dependent cellular uptake of the radioactive prodrug **C36** (Figure 19d), unveiling the preliminary hints of a potentially important glioblastoma inhibitor that should be investigated further. 

## 4. Conclusions and Future Perspectives

**Figure 20:**
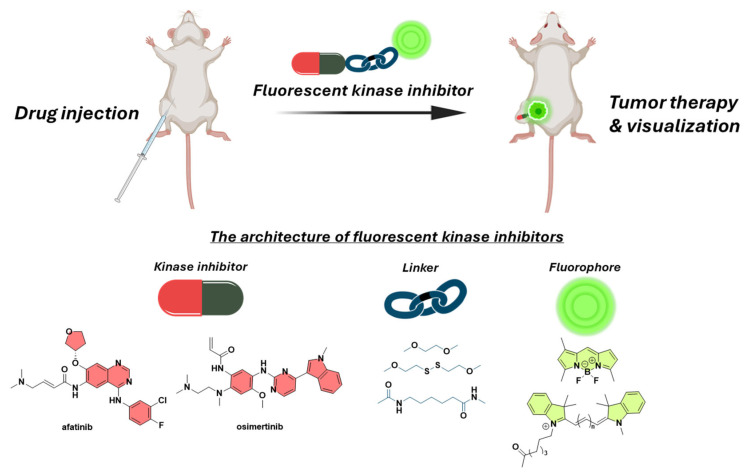
A schematic illustration highlighting the key components of a fluorescent drug conjugate: drug, linker, and fluorophore.

In summary, this review highlights the key design principles behind the development of fluorescent kinase inhibitors and their use as anticancer theranostic agents. By conjugating various small molecule kinase inhibitors with different fluorophores, researchers have generated several fluorescent kinase inhibitors with promising therapeutic as also diagnostic applications (Figure 20). 

Optimal efficacy requires careful consideration of all the components involved in the architecture of these compounds—drug, fluorophore, linker, and additional elements. Representative examples from the current literature have been discussed, paving the way for the development of new fluorescent inhibitors targeting the cancer microenvironment. Although this research area has grown in popularity in recent years, there is still a vast space to be explored. The limited number of reported conjugates emphasizes the need for further research to improve cancer theranostics. However, we anticipate that the continued advancements in NIR dyes and novel kinase inhibitors will provide the necessary tools to propel the field of fluorescent kinase inhibitors forward. 

## Figures and Tables

**Table 1:** Categorization of representative kinase inhibitors based on their targets.

FDA-Approved KINASE Inhibitors	Drug Target
Crizotinib, Ceritinib, Alectinib, Brigatinib, Lorlatinib	ALK
Bosutinib, Dasatinib, Imatinib, Nilotinib, Ponatinib	BCR–ABL
Vemurafenib, Dabrafenib, Encorafenib	B-Raf
Ibrutinib, Acalabrutinib, Zanubrutinib	BTK
Palbociclib, Sorafenib, Ribociclib Abemaciclib	CDK family
Crizotinib, Cabozantinib, Capmatinib	c-Met
Gefitinib, Erlotinib, Lapatinib, Vandetanib, Afatinib, Osimertinib, Dacomitinib	EGFR family
Neratinib, Tucatinib	ErbB2/HER2
Erdafitinib. Nintedanib, Pemigatinib	FGFR
Gilteritinib, Midostaurin	Flt3
Ruxolitinib, Tofacitinib, Baricitinib, Tofacitinib, Upadacitinib	JAK family
Trametinib, Binimetinib, Cobimetinib, Selumetinib	MEK1/2
Everolimus, Fedratinib, Sirolimus, Temsirolimus	Mtor
Axitinib, Gefitinib, Imatinib, Lenvatinib, Nintedanib, Pazopanib, Regorafenib, Sorafenib, Sunitinib, Avapritinib, Ripretinib	PDGFR α/β
Vandetanib, Cabozantinib, Pralsetinib, Selpercatinib	RET
Netarsudil	ROCK1/2
Entrectinib, Crizotinib	ROS1
Bosutinib, Dasatinib, Ponatinib, Vandetanib	Src family
Fostamatinib, R406	Syk
Larotrectinib	TRKA/B/C
Regorafenib, Pazopanib, Sorafenib, Axitinib, Lenvatinib, Nintedanib, Sunitinib, Cabozantinib, Vandetanib	VEGRF Family

**Table 2:** The inhibition effect of **C3a** and **C3b** on EGFR activity in A549 cells.

Compounds	IC_50_ (μM)
**C3a**	0.124
**C3b**	0.205
Erlotinib	5.182

**Table 3:** IC_50_ values for the erlotinib conjugates **C4a**, **C4b**, and phthalocyanine against HepG2 cancer cells with the light dose of 1.5 J/cm^2^.

Compounds	IC_50_ (mM)
**C4a**	0.01
**C4b**	0.04
Phthalocyanine	0.03
Erlotinib	*N* ^a^

^a^ Non-cytotoxic up to 0.5 μΜ.

**Table 4:** EC_50_ values of breast cancer cell lines and non-cancerous HEK293, 51D1, and 51D1.3 cell lines using WST-1 assay.

EC_50_ (nM)					
	MCF-7	MDA-MB-231	HEK293	51D1	51D1.3
**C12**	718.8 ± 74.1	871.6 ± 98.9	543.8 ± 5.9	265.0 ± 20.3	471.3 ± 61.2
**MH-148**	>2500	>2500	>2500	>2500	>2500
**Palbociclib**	>2500	>2500	>2500	>2500	>2500

**Table 5:** EC_50_ of the viability of the GBM cells and IC_50_ of the proliferation of the GBM cells.

	EC_50_ (nM)	IC_50_(nM)
**Crizotinib**	5600 ± 460	540 ± 160
**IR-786 iodide**	1680 ± 110	280 ± 70
**C13**	50 ± 20	4.7 ± 3.3

**Table 6:** IC_50_ and EC_50_ (GBM cells) values of dye, drug and conjugate **C22**. Data represent mean ± SEM. * = *p* < 0.05, *** = *p* > 0.005 relative to rucaparib.

	Compound		Compound with TMZ	
	EC_50_ (nM)	IC_50_ (nM)	EC_50_ (nM)	IC_50_ (nM)
**IR-786**	1735 ± 249	357 ± 39 ***	390 ± 45	120 ± 30 *
**C22**	128 ± 30	21 ± 4 ***	56 ± 6	20 ± 7 *
**Rucaparib**	>100,000	53,443 ± 473	>100,000	2262 ± 488
